# Thomson/Joule Power Compensation and the Measurement of the Thomson Coefficient

**DOI:** 10.3390/ma17184640

**Published:** 2024-09-21

**Authors:** Javier Garrido, José A. Manzanares

**Affiliations:** Departament de Termodinàmica, Universitat de Valencia, 46100 Burjassot, Spain; javier.garrido@uv.es

**Keywords:** thermoelectricity, Thomson coefficient, Thomson effect, Seebeck effect, energy balance

## Abstract

The energy transported by the electric current that circulates a thermoelectric element (TE) varies with position due to the Joule and Thomson effects. The Thomson effect may enhance or compensate the Joule effect. A method for measuring the Thomson coefficient of a TE is presented. This method is based on the total compensation of the Joule and Thomson effects. The electric current then flows without delivering power to the TE or absorbing power from it. For a TE, the global Thomson/Joule compensation ratio ${\bar{\Phi }}_{T/J}$ is defined as the ratio of the power absorbed by the current due to the Thomson effect and the power delivered by the current to the TE due to the Joule effect. It can be expressed as ${\bar{\Phi }}_{T/J}=I_{0}/I$, where *I* is the electric current and $I_{0}$ is the zero-power current, a quantity that is proportional to the average Thomson coefficient. When $I=I_{0}$, the Thomson effect exactly compensates the Joule effect and the net power delivered by the current to the TE is zero. Since the power delivered by the current is related to the temperature distribution, temperature measurements for currents around $I_{0}$ can be used as the basis for a measurement technique of the Thomson coefficient. With varying current, the difference between the temperature at the center of the TE and the mean temperature between its extremes reverses its sign at the zero-power current, $I=I_{0}$. This observation suggests the possibility of measuring the Thomson coefficient, but a quantitative analysis is needed. With calculations using the constant transport coefficients model for ${Bi}_{2}{\left({Te}_{0.94}{Se}_{0.06}\right)}_{3\;}$ and ${{\left({Bi}_{0.25}{Sb}_{0.75}\right)}_{2}Te}_{3}$, it is theoretically shown that a null temperature detector with a sensitivity of the order of 1 mK allows for the accurate determination of the Thomson coefficient.

## 1. Introduction

Thermoelectric generation and refrigeration have a long history of scientific and technological development, mainly due to the requirement for long-life electrical power sources [1]. Thermoelectric generators (TEGs) generate electric power from the electric current driven by a temperature difference. Thermoelectric coolers (TECs) drive a heat flow against a temperature difference by consuming electric power. Thermoelectric modules (TEMs) are relevant to developing sustainable processes and mitigating climate change. All aspects of thermoelectrics have experienced an unprecedented upsurge of activity [2,3], partly due to the increasing miniaturization of electronic circuits and sensors. For instance, small TEGs have drawn much attention as stand-alone, energy-harvesting power sources for wearable electronics [4,5].

The Thomson effect is the rate of energy supply to the conductor by the electric current due to the presence of a temperature gradient [6]. Although early studies considered it to be not of primary importance or even negligible [1], the Thomson effect needs to be accurately described in the energy balance in order to optimize the efficiency of both TEGs and TECs [7,8,9,10,11,12]. Since the energy transported by the electric current varies with position along the conductor, the Thomson effect can be described as electric power delivered by the current to the conductor. The rationale that the Thomson effect is electrical power lies in the distinction of energy observables. The energy rate is determined by the observable heat rate $\dot{Q}$ and the electrical power P supplied by the current, $\dot{U}=\dot{Q}+P$ [6]. The observable heat rate only includes the divergence of the Fourier flux density. The power that the electric current delivers to a conductor has two components: Joule and Thomson. Joule power is always positive, while Thomson power has no definite sign.

Lan et al. ranked the Thomson effect as the second most important factor influencing the efficiency of TEGs [13]. Luo et al. developed a model, including the Thomson effect, to study the parameters characterizing the behavior of TEGs and TECs [14]. Zhang et al. showed that the Thomson effect affects heat production, heat transfer, and energy conversion in TEGs. The output power and efficiency are increased by 15.6% and 8.9%, respectively, due to the Thomson effect [15]. Chengjian et al. studied the influence of the Thomson effect in annular TEGs [16]. Chen et al. studied the influence of the Thomson effect in the combined TEG–thermoelectric heat pump [17]. Bakan et al., after stating that electrical charges in a conductor carry different types of electrical power—basic, kinetic, and chemical—studied the conditions that the electrical pulses must meet to suppress the Thomson heat [18]. Qiu et al. analyzed the influence of the Thomson effect on TECs by evaluating the heat rates, instead of the (more common) heat fluxes arriving at the ends of each thermoelectric element (TE) [19]. Ponnusamy et al. analyzed how the Thomson heat influences the bending of the temperature distribution in the TEs of a TEG [20]. Ruiz-Ortega et al. studied an electric pulse in a double-stage TEC. They considered that the significant drop in temperature on the cold side depends strongly on the Thomson effect. The maximum COP increases by 75% when the Thomson effect is considered [21]. Wielgosz et al. developed two numeric models on the thermoelectric behavior of unicouples in TEGs. In the heat balance, apart from the Joule and Thomson heats, they added two new terms, the Peltier and Bridgman heats [22]. Gong et al. highlighted the influence of the Thomson effect on the cooling capacity of TECs [23]. Sun et al. explored the role of the Thomson effect on micro-thermoelectric coolers. When considering the Thomson effect, the minimum cooling temperature can be reduced by 4.1 K [24]. Chen et al. included the Thomson effect when analyzing the efficiency of the photovoltaic cell–TEG coupling [25]. Cui et al. included the Thomson effect in the study of the delamination and thermoelectric performance of *p-n* junctions [26]. Shi et al. studied the influence of the Thomson effect in a two-dimensional thermoelectric plate. They deduced that the normalized thermoelectric conversion efficiency would increase by 10% when the Thomson effect was considered [27]. Lafaurie et al. described a non-linear Thomson effect produced in a TE by a transient current [28]. Chiva et al. studied the temperature distribution that the Thomson effect generates in a TE [29].

In all these references [7,8,9,10,11,12,13,14,15,16,17,18,19,20,21,22,23,24,25,26,27,28,29], the Thomson energy was described as heat generated by the current, but it can be better described as electrical power that the current supplies to the material [6]. In a TE, the current develops two types of power: Joule power and Thomson power [6]. The Joule power is always a positive quantity, while the Thomson power does not have a definite sign [1,6]. In the present work, the possible compensation of the positive Joule power with negative values of the Thomson power are analyzed. Once the Thomson/Joule compensation has been defined, three types of compensation will be analyzed: enhancement, undercompensation, and overcompensation. Overcompensation means that, due to the Thomson effect, the current absorbs energy from the conductor at a higher rate than that of delivery due to the Joule effect. This occurs for currents in the range $0<I/I_{0}<1$, where $I_{0}$ is the zero-power current. For many materials, this range is narrow and of moderate practical interest. However, this range widens when the Thomson coefficient increases because $I_{0}$ is proportional to it. For recent materials exhibiting a giant Thomson effect, this range is of utmost interest [7,30]. When the T/J compensation is exact, the current flows without absorbing or delivering energy to the conductor. 

The traditional methods for the measurement of the Thomson coefficient in metallic samples are based on establishing the temperature gradient in the sample and detecting temperature changes [31]. To make it accurate at high temperatures, the method was developed by Roberts when establishing his absolute scale of thermoelectricity [32]. More recent methods, especially those suitable for thin films, compare the change in the temperature at the central point of the sample under the flow of DC and AC electric currents [33]. One of the main aims of this work is to show that the exact T/J compensation can serve as the basis for a measurement technique of the Thomson coefficient.

Another aim is to analyze the influence of the Thomson effect on the parameters that characterize the behavior of a TEM. The cooling capacity ${\dot{Q}}_{s\to c}$ of a TEC and the efficiency η of a TEG depend on the Thomson/Joule compensation in the semiconductor legs [34]. The Thomson coefficients do not appear explicitly in the expressions of $P_{out}$ and $P_{in}$ [35]. In a thermoelectric circuit, the compensation of the Joule power is not developed by the Thomson power but by the Seebeck power. This Seebeck power is the sum of the Thomson powers of the TEs of the circuit plus the Peltier powers of the junctions between the TEs.

## 2. Theory

### 2.1. Energy Balance in the Observable Formulation

In the observable formulation, the non-equilibrium states are locally characterized by the temperature gradient ∇T and the electric current density i [6,36,37,38,39,40]. The transport processes of electrical charge and energy cannot be separated [41]. The Seebeck coefficient S of the conductor is defined via the transport equation for the charge

(1) $$\rho i=\frac{1}{e}\nabla \mu -S\nabla T,$$

where ρ is its electrical resistivity, μ is the electrochemical potential of the electrons, and *e* (>0) is the elementary charge. The energy flux density is

(2) $$j_{u}=-\kappa \nabla T+\left(\Pi -\frac{\mu }{e}\right)i$$

where κ is the thermal conductivity and Π is the Peltier coefficient. The first term in Equation (2) is the heat flux density as described by Fourier’s law

(3) $$j_{q}=-\kappa \nabla T$$

i.e., the diffusive contribution due to ∇T. The second term in Equation (2) is the contribution due to i, and we denote it as the work flux density

(4) $$-j_{w}=\left(\Pi -\frac{\mu }{e}\right)i$$

using the sign convention $j_{u}=j_{q}-j_{w}$ [1]. The rate of increase in the internal energy of a finite region V of the conductor due to the flux through the surface ∂V enclosing it is $-{∯}_{\partial V}j_{u}\cdot dA=-{∭}_{V}\nabla \cdot j_{u}d^{3}r$, where dA is a surface element vector pointing outside V and we have used the divergence theorem. For an infinitesimal volume element δV, its rate of increase in the internal energy can be written as $\delta \dot{U}=-\nabla \cdot j_{u}\delta V$. It is the sum of two contributions due to ∇T and i, $\delta \dot{U}=\delta \dot{Q}+\delta P$, where $\delta \dot{Q}=-\nabla \cdot j_{q}\delta V$ is the heating power (positive when energy flows to δV due to ∇T from its surroundings) and $\delta P=\nabla \cdot j_{w}\delta V$ is the power delivered to δV by the electric current. Under steady-state conditions, the internal energy of δV does not change with time and $\delta \dot{Q}+\delta P=0$. For instance, when the Joule effect dominates, the energy delivered by the current to δV is positive, and the same quantity of heat flows by conduction to its surroundings, $\delta P=-\delta \dot{Q}>0$.

The Seebeck, Peltier, and Thomson effects are interrelated [40,41]. Under steady-state conditions, using Equations (1) and (2) and the conservation of charge ∇·i=0, the energy balance equation $\nabla \cdot j_{u}=0$ can be presented as

(5) $$0=\nabla \cdot \left(\kappa \nabla T\right)+\rho i^{2}-\tau i\cdot \nabla T,$$

where τ=dΠ/dT−S is the Thomson coefficient. The power $\delta P=\nabla \cdot j_{w}\delta V$ that enters an infinitesimal volume element δV due to i is the sum contribution of the Joule effect $\delta P_{J}=\rho i^{2}\delta V>0$ and the Thomson effect $\delta P_{T}=-\tau i\cdot \nabla T\delta V$, as the divergence of the work flux density is

(6) $$\nabla \cdot j_{w}=\rho i^{2}-\tau i\cdot \nabla T.$$

The nature of the Joule and Thomson contributions as *powers* delivered by the current is further discussed in Appendix A. The sign of the Thomson power $\delta P_{T}$ reverses with the direction of the current density. When the Thomson power is positive, it enhances the Joule effect. A negative Thomson power $\delta P_{T}<0$ means that the electric current absorbs power from the TE, thus compensating the Joule effect. In most cases, this compensation is only partial, so that the net effect is a power delivery to δV due to i, δP>0. However, in some cases the negative Thomson power can overcompensate the positive Joule power and δP<0. The exact compensation between the Thomson and Joule effects that can occur under some experimental conditions enables the measurement of the Thomson coefficient.

### 2.2. Thomson/Joule Power Compensation in a Thermoelectric Element

Consider a bar-shaped, thermoelectric element (TE) of cross-section A and length L with thermally insulated lateral surfaces. The position coordinate x increases from the cold boundary, with temperature $T_{c}=T\left(x=0\right)$, to the hot one, $T_{h}=T\left(x=L\right)$, so that dT/dx>0 for all or most of the bar. The electric current I=iA is positive when it flows in the positive *x* direction. The one-dimensional form of the steady-state, local energy balance equation, Equation (5), is

(7) $$0=\frac{d}{dx}\left(\kappa \frac{dT}{dx}\right)+\rho {\left(\frac{I}{A}\right)}^{2}-\tau \frac{I}{A}\frac{dT}{dx}.$$

The heat flow that enters a volume δV=Aδx of the bar from their neighboring volume elements due to their temperature difference is $\delta \dot{Q}=\delta Vd(\kappa dT/dx)/dx$. The power that enters δV due to the electric current *I* is the sum of the Joule and Thomson contributions, $\delta P_{J}=\delta V\rho (I/A{)}^{2}$ and $\delta P_{T}=-\delta V\tau (I/A)dT/dx$, respectively.

In terms of the local Thomson/Joule (T/J) compensation ratio

(8) $$\Phi_{T/J}\left(x\right)=-\frac{\delta P_{T}}{\delta P_{J}}=\frac{\tau A}{\rho I}\frac{dT}{dx},$$

Equation (7) can be presented as

(9) $$\frac{d}{dx}\left(\kappa \frac{dT}{dx}\right)=\rho {\left(\frac{I}{A}\right)}^{2}\left[\Phi_{T/J}(x)-1\right].$$

Equation (9) shows that the T/J compensation is related to the curvature of $T\left(x\right)$. The ratio $\Phi_{T/J}\left(x\right)$ depends on dT/dx, I/A and the transport coefficients τ and ρ. Compensation requires $\Phi_{T/J}>0$ and, hence, $\delta P_{T}<0.$ Thus, compensation requires τ>0 when $I\left(dT/dx\right)>0$. Overcompensation $\Phi_{T/J}(x)>1$ implies $d\left(\kappa dT/dx\right)/dx>0$. Undercompensation $\Phi_{T/J}(x)<1$ and enhancement $\Phi_{T/J}(x)<0$ imply $d\left(\kappa dT/dx\right)/dx<0$.

In a TE of length L, the Joule and Thomson powers are $P_{J}\left(I\right)=I^{2}R$ and $P_{T}\left(I\right)=-I\bar{\tau }∆T$, where $R=\left(1/A\right)\int_{0}^{L}\rho dx$ is the electrical resistance, $\bar{\tau }=\left(1/∆T\right)\int_{T_{c}}^{T_{h}}\tau dT$ is the average Thomson coefficient, and $∆T=T_{h}-T_{c}>0$. The power delivered by the current is

(10) $$P\left(I\right)=I^{2}R-I\bar{\tau }∆T=\left(1-I_{0}/I\right)I^{2}R,$$

where $I_{0}=\bar{\tau }∆T/R$ is the zero-power current, $P\left(I_{0}\right)=0.$ Exact Thomson/Joule compensation occurs when $I=I_{0}$, because the power that enters the TE due to the Joule effect is then equal to the power that leaves the TE due to the Thomson effect, $P_{J}\left(I_{0}\right){=-P}_{T}\left(I_{0}\right)$. Since ∆T>0 and R>0, $I_{0}$ has the same sign as the average Thomson coefficient $\bar{\tau }$.

On a global analysis, the Thomson/Joule compensation can be quantified by the ratio

(11) $${\bar{\Phi }}_{T/J}=-\frac{P_{T}\left(I\right)}{P_{J}\left(I\right)}=\frac{\bar{\tau }I∆T}{I^{2}R}=\frac{I_{0}}{I}.$$

**Figure 1:**
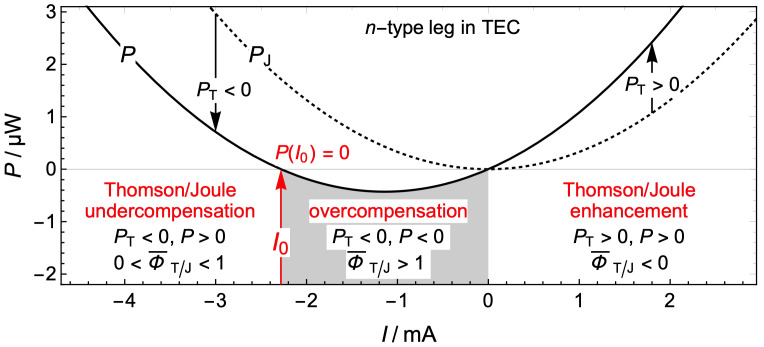
Global Thomson/Joule undercompensation, overcompensation, and enhancement occur in different ranges of the electric current I. The electric power $P\left(I\right)$ (solid curve) delivered by the current to the TE is the sum of the Joule power $P_{J}=I^{2}R\;$(dotted curve) and the Thomson power $P_{T}=-I\bar{\tau }∆T=-I_{0}IR$. For the zero-power current $I=I_{0}$, the Thomson effect exactly compensates the Joule effect and the net power delivered by the current is zero, $P\left(I_{0}\right)=0$.

When ${\bar{\Phi }}_{T/J}<0$, the Thomson effect enhances the Joule effect $\left(P>I^{2}R\right)$ (Figure 1). When $0<{\bar{\Phi }}_{T/J}<1$, the Thomson effect undercompensates the Joule effect $\left(I^{2}R>P>0\right)$. When ${\bar{\Phi }}_{T/J}>1$ and P<0, overcompensation occurs, which requires currents in the range $0<I/I_{0}<1$. This range widens when $\left|\bar{\tau }\right|$ increases because $I_{0}=\bar{\tau }∆T/R\propto \bar{\tau }$. For many materials, this range is narrow and of moderate practical interest. However, for recent materials exhibiting a giant Thomson effect, this range is of utmost interest [7,30].

### 2.3. Measurements of the Thomson Coefficient Based on the Constant Transport Coefficients Model

The temperature distribution $T\left(x\right)$ in the TE is the solution of Equation (7) [20]. When κ, ρ and τ are assumed to be constants, this solution is

(12) $${T\left(x\right)-T}_{c}=\Delta T\frac{x}{L}+\frac{P}{\tau I}\left[\frac{x}{L}-\frac{exp\left(\tau Ix/KL\right)-1}{exp\left(\tau I/K\right)-1}\right],$$

where K=κA/L is the thermal conductance. The difference δT(I) between the temperature $T\left(L/2\right)$ at the center x=L/2 and the mean temperature $T_{m}=\left(T_{h}+T_{c}\right)/2$ is [36]

(13) $$\delta T(I)={T\left(L/2\right)-T}_{m}=\frac{P}{2\tau I}tanh\frac{\tau I}{4K}\approx \frac{P}{8K}.$$

The approximation is valid when $\left|\tau I\right|\ll K$, which is rather common. That is, while the variation of $T\left(x\right)$ with the electric current I can be complicated in general, the variation of δT(I) is rather simple. This explains why several measurement methods of the Thomson coefficient τ make use of δT(I). 

The power is given by Equation (10), $P=P_{J}\left(I\right)+P_{T}\left(I\right)=\left(1-I_{0}/I\right)I^{2}R$. When reversing the direction of the electric current, $P_{T}\left(I\right)=-I\bar{\tau }∆T=-II_{0}R$ changes sign and $P_{J}\left(I\right)$ remains invariant. Therefore, δT is sensitive to the direction of the current and the measurements of δT(I) and δT(−I) can be combined to evaluate τ, as in Nettleton’s method [31]. First, we note that the Thomson coefficient τ is proportional to $\delta T(I)-\delta T(-I)\approx P_{T}\left(I\right)/4K$. Second, we note that $\delta T(I)+\delta T(-I)\approx P_{J}\left(I\right)/4K$. In fact, in the absence of the Thomson effect, $\delta T_{\tau =0}(I)=P_{J}\left(I\right)/8K$, which is independent of the direction of the current. A problem related to the determination of τ from δT(I)−δT(−I) is that the measurement of the thermal conductance K is required and difficult. This problem can be circumvented by eliminating K through the evaluation of

(14) $$\frac{\delta T(I)-\delta T(-I)}{\delta T(I)+\delta T(-I)}\approx \frac{P_{T}\left(I\right)}{P_{J}\left(I\right)}=-{\bar{\Phi }}_{T/J}=-\frac{\bar{\tau }I∆T}{I^{2}R}\;.$$

Experimentally, a combination of DC currents and AC currents can serve this purpose [33].

Two currents, $I_{1}$ and $-I_{2}$, that differ in magnitude and direction can produce the same δT at x=L/2 [32]. From Equation (13), the condition $\delta T(I_{1})=\delta T(-I_{2})$ is equivalent to $P_{J}\left(I_{1}\right)+P_{T}\left(I_{1}\right)=P_{J}\left(I_{2}\right)-P_{T}\left(I_{2}\right)$, which can easily be transformed to $I_{0}=\tau ∆T/R=I_{1}-I_{2}$ by using Equation (10). The expression $\tau =(I_{1}-I_{2})R/∆T$ constitutes the basis of Roberts’ method to measure the Thomson coefficient [31,32]. The exact T/J compensation ${\bar{\Phi }}_{T/J}=1$ at the zero-power current $I_{0}$ implies $P(I_{0})=0$ and $\delta T(I_{0})=0$ and it can also be used as another method to measure the Thomson coefficient, as discussed below.

The curvature $d^{2}T/dx^{2}\approx -P/KL^{2}$ of the temperature distribution is determined by the power $P=\left(1-I_{0}/I\right)I^{2}R$ delivered by the current. It vanishes in the absence of current and at the zero-power current ${I=I}_{0}$, when exact compensation between the Thomson and Joule effects occurs. In these two cases, the temperature distribution is linear and the temperature at the center x=L/2 of the TE is $T_{m}=\left(T_{c}+T_{h}\right)/2$. When ${I/I}_{0}>0$ increases from 0, the difference $T\left(L/2\right)-T_{m}\approx P/8K={(I}^{2}R/8K)\left(1-I_{0}/I\right)$ first takes negative values (due to overcompensation), becomes zero at ${I=I}_{0}$ (compensation), and is positive for ${I/I}_{0}>1$ (undercompensation or enhancement).

From Equation (12), the relation between the local and global T/J compensation ratios is

(15) $$\frac{1-\Phi_{T/J}\left(x\right)}{1-{\bar{\Phi }}_{T/J}(I)}=\frac{\tau I/K}{e^{\tau I/K}-1}e^{\tau Ix/KL}\approx 1+\frac{\tau I}{K}\left(\frac{x}{L}-\frac{1}{2}\right),$$

where $\left|\tau I\right|\ll K$ has been used in the approximation. Thus, at the center x=L/2 of the TE, the local T/J compensation ratio is approximately equal to the global T/J compensation ratio. At any position *x*, Equation (15) implies that $1-\Phi_{T/J}\left(x\right)$ and $1-{\bar{\Phi }}_{T/J}(I)$ have the same sign because $(\tau I/K)/\left(e^{\tau I/K}-1\right)>0$. From Equations (9) and (15), the temperature distribution is the same for zero current and for the zero-power current, because ${\bar{\Phi }}_{T/J}(I_{0})=1$ implies $\Phi_{T/J}\left(x\right)=1$ (in the constant transport coefficients model). If the Thomson effect globally overcompensates the Joule effect, ${\bar{\Phi }}_{T/J}(I)>1$ when $0<I/I_{0}<1$, then overcompensation occurs locally at every position. Similarly, if the Thomson effect globally undercompensates or enhances the Joule effect, ${\bar{\Phi }}_{T/J}(I)<1$, then undercompensation ${(0<\Phi }_{T/J}\left(x\right)<1)$ or enhancement ${(\Phi }_{T/J}\left(x\right)<0)$ occurs at every position.

### 2.4. Energy Balances and Thomson/Joule Compensation in TEGs and TECs

**Figure 2:**
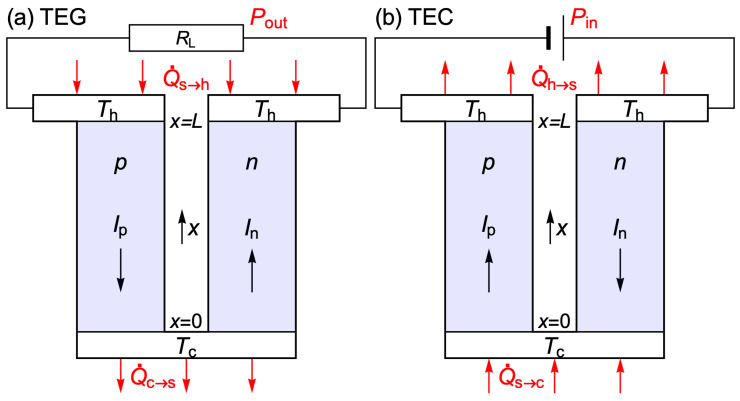
Basic unit of a thermoelectric module (TEM): *n*-type and *p*-type legs and metal connectors at cold $\left(T_{c}\right)$ and hot ${(T}_{h})$ temperatures. Under steady-state conditions, the energy rate (between the TEM and its surroundings) is zero. (**a**) As a thermoelectric generator (TEG), the net heat flow to the connectors, ${\dot{Q}}_{s\to h}-{\dot{Q}}_{c\to s}$, is equal to the power $P_{out}$ delivered to the load resistance $R_{L}$. The electrons and holes generated at the hot connector flow towards the cold connector, where they recombine. (**b**) As a thermoelectric cooler (TEC), the power input $P_{in}$ from the external source is equal to the net heat flow from the connectors to its surroundings, ${\dot{Q}}_{h\to s}-{\dot{Q}}_{s\to c}$. The electrons and holes generated at the cold connector flow towards the hot connector, where they recombine.

Consider the basic unit of a thermoelectric module (TEM), i.e., the *n*-type and *p*-type legs and the connectors at different cold and hot temperatures (Figure 2). In the absence of external power sources, when the circuit is closed, the electric current flows in the direction that tends to reduce the difference $T_{h}-T_{c}$, thus driving the system towards thermal equilibrium. The scalar product of Equation (4) with the current density i leads to $(1/e)Id\mu ={(\rho /A)I}^{2}dx+ISdT$, where the current I is positive when it flows in the direction of increasing temperature and negative otherwise. Its integration around the closed circuit shows that the driving force for the electric current is $\int_{T_{c}}^{T_{h}}\left(S_{p}-S_{n}\right)dT>0$. The electrochemical potential of the electrons is a continuous function of position, and therefore ∮Idμ=0. Hence, the Joule power $P_{J}=(I^{2}/A)\oint \rho dx>0$ must be provided by the Seebeck power, $P_{S}=\oint ISdT<0$. We can refer to the energy balance $P_{J}=-P_{S}$ as a Seebeck/Joule compensation for the closed circuit (Appendix B). That is, the power delivered to the conductors by the current due to the Joule effect is the same as the power $-P_{S}>0$ absorbed by the current from the conductors due to the Seebeck effect.

A thermoelectric generator (TEG) takes advantage of the tendency of the current to flow in the direction that tends to reduce the difference $T_{h}-T_{c}$ to drive current through an external load. When a resistance $R_{L}$ is connected to the terminals of the TEG, the current delivers an output power $P_{out}=I^{2}R_{L}$ to the load resistor. The total Joule power, $P_{out}+P_{J,TEM}$, where $P_{J,TEM}=I^{2}R_{TEM}$ and $R_{TEM}$ is the electrical resistance of the TEM, is provided by the Seebeck power

(16) $$P_{out}+I^{2}R_{TEM}=-P_{S}=\left|I\right|\int_{T_{c}}^{T_{h}}\left(S_{p}-S_{n}\right)dT>0$$

In the evaluation of this Seebeck power $P_{S}=\oint ISdT$, which drives the current in the TEG, the current in the *p*-type leg is $I_{p}=-\left|I\right|<0$ because it flows in the direction of decreasing temperature, and the current in the *n*-type leg is $I_{n}=\left|I\right|>0$ (Figure 2a). If thermal reservoirs (s) maintain constant junction temperatures, then the TEG reaches the steady state when the energy rate (Figure 2a) is zero,

(17) $$P_{out}=-P_{TEM}={\dot{Q}}_{TEM}={\dot{Q}}_{s\to h}-{\dot{Q}}_{c\to s}$$

where $P_{TEM}$ is the electric power received by the TEM. The efficiency is $\eta =P_{out}/{\dot{Q}}_{s\to h}$.

A thermoelectric cooler (TEC) uses an external power source to drive the electric current through the module in the direction that tends to increase the difference $T_{h}-T_{c}$; that is, $I_{p}=\left|I\right|>0$ in the *p*-type leg, as it flows in the direction of increasing temperature, and $I_{n}=-\left|I\right|<0$ in the *n*-type leg (Figure 2b). The Seebeck power is then positive, $P_{S}=\oint ISdT=\left|I\right|\int_{T_{c}}^{T_{h}}\left(S_{p}-S_{n}\right)dT>0$, as the Joule power. The power that the current delivers, by the Joule and Seebeck effects, to the conductors of the closed circuit must be provided externally. By integration of $(1/e)Id\mu ={(\rho /A)I}^{2}dx+ISdT$ around the circuit with the external source, it is concluded that the power provided by this source to drive the current through the TEM is

(18) $$P_{in}=I^{2}R_{TEM}+\left|I\right|\int_{T_{c}}^{T_{h}}\left(S_{p}-S_{n}\right)dT>0.$$

Under steady-state conditions, the energy rate is zero, ${\dot{Q}}_{TEM}+P_{TEM}=0$ (Figure 2b), and

(19) $$P_{in}=P_{TEM}={\dot{Q}}_{h\to s}-{\dot{Q}}_{s\to c}>0.$$

The cooling capacity of the TEC is ${\dot{Q}}_{s\to c}>0$, i.e., the rate of energy extraction from the cold thermal reservoir (s).

The cooling capacity ${\dot{Q}}_{s\to c}$ of a TEC and the efficiency η of a TEG depend on the T/J compensation in the semiconductor legs [34]. The heat rate in the cold and hot connectors and the powers delivered to them by the electric current are

(20) $${\dot{Q}}_{c}=A\left[{\left(\kappa_{p}\frac{dT_{p}}{dx}\right)}_{x=0}+{\left(\kappa_{n}\frac{dT_{n}}{dx}\right)}_{x=0}\right]+{\dot{Q}}_{s\to c}$$

(21) $$P_{c}=I^{2}R_{c}-\left(\Pi_{pc}-\Pi_{nc}\right)I$$

(22) $${\dot{Q}}_{h}={\dot{Q}}_{s\to h}-A\left[{\left(\kappa_{p}\frac{dT_{p}}{dx}\right)}_{x=L}+{\left(\kappa_{n}\frac{dT_{n}}{dx}\right)}_{x=L}\right]$$

(23) $$P_{h}=I^{2}R_{h}-\left(\Pi_{ph}-\Pi_{nh}\right)I,$$

where $R_{i}$ (i=c,h) is the electrical resistance of the cold or hot connector, and $\Pi_{ij}$ is the Peltier coefficient of leg *i* (i=p,n) at temperature *j* (j=h, c). Under steady-state conditions, ${\dot{Q}}_{i}+P_{i}=0$, Equations (19)–(23) show that

(24) $${\dot{Q}}_{s\to c}=\left(\Pi_{pc}-\Pi_{nc}\right)I-I^{2}R_{c}-A\left[{\left(\kappa_{p}\frac{dT_{p}}{dx}\right)}_{x=0}+{\left(\kappa_{n}\frac{dT_{n}}{dx}\right)}_{x=0}\right]$$

(25) $${\dot{Q}}_{s\to h}=\left(\Pi_{ph}-\Pi_{nh}\right)I-I^{2}R_{h}+A\left[{\left(\kappa_{p}\frac{dT_{p}}{dx}\right)}_{x=L}+{\left(\kappa_{n}\frac{dT_{n}}{dx}\right)}_{x=L}\right]$$

are affected by the Thomson effect, because the T/J compensation is related to the curvature of the temperature distribution in the legs.

The output power $P_{out}$ of a TEG and the input power $P_{in}$ of a TEC involve the integral $\int_{T_{c}}^{T_{h}}\left(S_{p}-S_{n}\right)dT$, Equations (16) and (18). In agreement with previous results [35], the Thomson coefficients practically do not affect the powers $P_{out}$ and $P_{in}$.

## 3. Results

### 3.1. Thomson Effect in a TEC Using the Constant Transport Coefficients Model

The constant transport coefficients model is used to analyze the T/J compensation in a TEC. We consider legs of length L=10 mm and section area $A=0.50\times 0.50\;{mm}^{2}$, with temperatures $T_{c}=290\;K$ and $T_{h}=310\;K$ at their boundaries. The *n*-type leg is ${Bi}_{2}{\left({Te}_{0.94}{Se}_{0.06}\right)}_{3}$, with $\kappa_{n}=1.643\;{W\;m}^{-1}K^{-1}$, $\rho_{n}=8.239\;\mu \Omega \;m$, and $\tau_{n}=-37.60\;\mu V\;K^{-1}$ as typical values in this temperature range. The *p*-type leg is ${{\left({Bi}_{0.25}{Sb}_{0.75}\right)}_{2}Te}_{3}$, with $\kappa_{p}=1.472\;{W\;m}^{-1}K^{-1}$, $\rho_{p}=8.826\;\mu \Omega \;m$, and $\tau_{p}=102.6\;\mu V\;K^{-1}$ [42,43,44]. The corresponding electrical resistances and thermal conductances are $R_{n}=0.330\;\Omega$, $R_{p}=0.353\;\Omega$, $K_{n}=41.1\;\mu W\;K^{-1}$, and $K_{p}=36.8\;\mu W\;K^{-1}$. The material performance of these products can vary significantly depending on the strategy that has been developed in their production [45].

**Figure 3:**
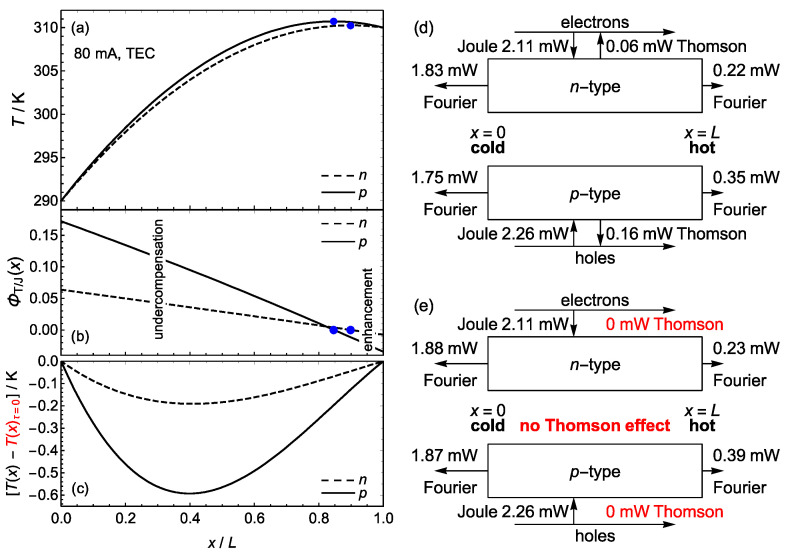
(**a**) Temperature distribution $T\left(x\right)$ for a current of 80 mA in a TEC, Equation (12). (**b**) Local T/J compensation ratio $\Phi_{T/J}\left(x\right)$, Equation (8). Undercompensation occurs between the cold boundary and the positions (marked with points) of maximum temperature. Enhancement occurs between those positions and the hot boundary. (**c**) The Thomson effect affects the temperature distribution. In the ordinate axis, the difference between the temperature with and without Thomson effect is represented. With Thomson effect, the temperature $T\left(x\right)$ is lower than without Thomson effect. (**d**) Global balances of the Joule power, the Thomson power and the Fourier fluxes $J_{q}=-\kappa A\left(dT/dx\right)$ in the legs. (**e**) In the absence of Thomson effect, the Fourier fluxes are increased because so are the temperature gradients at the cold and hot boundaries.

A relatively small electric current $I_{p}=-I_{n}=80\;mA$ delivers to the conductor the powers $P_{n}=(2.11-0.06)\;mW=2.05\;mW$ and $P_{p}=(2.26-0.16)\;mW=2.10\;mW$ (Figure 3d). The Fourier fluxes at the boundaries determine the heating rates ${\dot{Q}}_{n}=-(1.83+0.22)\;mW=-2.05\;mW$ and ${\dot{Q}}_{p}=-(1.75+0.35)\;mW=-2.10\;mW$.

Since the zero-power currents are $I_{0,n}=\tau_{n}\Delta T/R_{n}=-2.28\;mA$ and $I_{0,p}=\tau_{p}\Delta T/R_{p}=5.81\;mA$, global T/J undercompensation occurs (see Figure 1), as indicated by ratios ${\bar{\Phi }}_{T/J,n}=I_{0,n}/I_{n}=0.0285$ and ${\bar{\Phi }}_{T/J,p}=I_{0,p}/I_{p}=0.0726$. On a local basis, the T/J compensation is proportional to the temperature gradient, $\Phi_{T/J}\left(x\right)=(\tau A/\rho I)dT/dx$, Equation (8). In both legs, undercompensation $\left(0<\Phi_{T/J}\left(x\right)<1\right)$ occurs between the cold boundary and the position of maximum temperature, and enhancement $\left(\Phi_{T/J}\left(x\right)<0\right)$ between that position and the hot boundary (Figure 3b).

The Thomson effect affects the cooling capacity ${\dot{Q}}_{s\to c}$ of a TEC through the temperature gradient at the cold junction (see Equation (24)). From Equations (10) and (12), this gradient is

(26) $${\left.\frac{dT}{dx}\right|}_{x=0}\approx \frac{\Delta T}{L}+\frac{I^{2}R}{2KL}-\frac{\bar{\tau }I}{2K}\frac{\Delta T}{L}.$$

Since $\tau_{n}I_{n}>0$ and $\tau_{p}I_{p}>0$ in a TEC, the Thomson effect reduces the gradient ${\left.dT/dx\right|}_{x=0}$ (compare Figure 3d,e). Using the above values of the transport coefficients, ${\left.dT/dx\right|}_{x=0}$ can be evaluated in the *n*-type leg as 4.57 K/mm for $\tau_{n}=0$ and 4.49 K/mm for $\tau_{n}=-37.60\;\mu V\;K^{-1}$, and in the *p*-type leg as 5.07 K/mm for $\tau_{p}=0$ and 4.87 K/mm for $\tau_{p}=102.6\;\mu V\;K^{-1}$. From Equation (24) and the temperature distributions in Figure 3a,c, it is concluded that ${\dot{Q}}_{s\to c}$ increases by 1.2% due to the Thomson effect.

### 3.2. The Effect of the Thomson Coefficient on the Thomson/Joule Compensation

**Figure 4:**
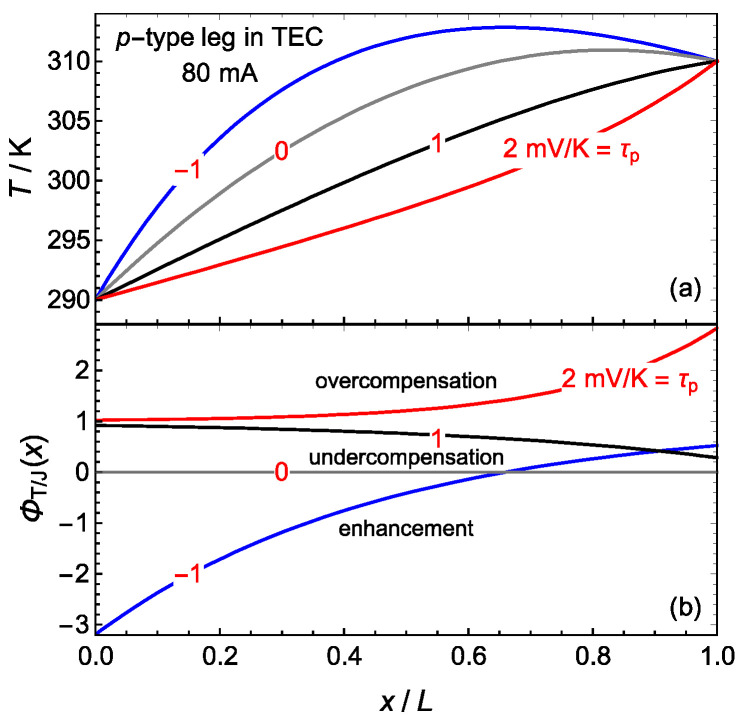
(**a**) Temperature distributions and (**b**) local T/J compensation ratios for a current of 80 mA in the *p*-type leg in a TEC and hypothetical values of the Thomson coefficient: −1.0, 0.0, 1.0 and $2.0\;mVK^{-1}$.

The T/J compensation increases when the Thomson coefficient τ of the material is increased [7]. However, the dependence of the local T/J compensation $\Phi_{T/J}(x)$ on τ is not trivial because $\Phi_{T/J}(x)$ is proportional to the product of the τ and the temperature gradient, Equation (8), and any change in τ also modifies this gradient. For instance, Figure 3c shows that the temperature gradient at the cold junction decreases in both semiconductor legs due to the Thomson effect. In order to analyze the dependence of the local T/J compensation $\Phi_{T/J}(x)$ on τ , we show the results obtained for semiconductors ${Bi}_{2}{\left({Te}_{0.94}{Se}_{0.06}\right)}_{3\;}$ and ${{\left({Bi}_{0.25}{Sb}_{0.75}\right)}_{2}Te}_{3}$ with hypothetical values of the Thomson coefficient: −1.0, 0.0, 1.0 and $2.0\;mV\;K^{-1}$ [23,29]. The current is I=80 mA and the boundary temperatures are $T_{c}=290\;K$ and $T_{h}=310\;K$. Figure 4 presents the results for the *p*-type leg in a TEC using the constant transport coefficients model. Very similar values are obtained for *n*-type leg. For these values of the Thomson coefficient, the global T/J compensation ratios ${\bar{\Phi }}_{T/J}(I)$ are, respectively, −0.708, 0, 0.708 and 1.416, corresponding to enhancement, absence of compensation, undercompensation and overcompensation. Figure 4b shows that, for $\tau =-1.0\;mV\;K^{-1}$, the local T/J compensation ratio $\Phi_{T/J}(x)$ is negative (corresponding to enhancement) for x/L<0.65 and positive but less than one (undercompensation) for x/L>0.65. For the other values of τ, the local T/J compensation ratio $\Phi_{T/J}(x)$ corresponds to the global T/J ratio ${\bar{\Phi }}_{T/J}(I)$.

It is noteworthy that doubling the Thomson coefficient from 1.0 to $2.0\;mV\;K^{-1}$ does not double the local T/J compensation ratio because the Thomson coefficient also modifies the temperature gradient. At the cold junction x=0, the temperature gradient decreases when doubling the Thomson coefficient from 1.0 to $2.0\;mV\;K^{-1}$ and $\Phi_{T/J}(0)$ only increases by 10%. The dependence of the cooling capacity ${\dot{Q}}_{s\to c}$ on the Thomson coefficient becomes evident when examining different temperature distributions (Figure 4a). The Fourier term in Equation (24), $\kappa_{p}A{\left(dT_{p}/dx\right)}_{x=0}$, reduces in value as the Thomson coefficient increases. Intercalation of heat dissipative elements in the leg gives similar results [46].

### 3.3. The Thomson/Joule Compensation Enables the Measurement of the Thomson Coefficient

In a TE, the global T/J compensation varies with the current I (Figure 1). When ${I=I}_{0}$ and ${\bar{\Phi }}_{T/J}=1$, the Thomson power exactly compensates the Joule power and the current circulates without absorbing or delivering energy to the conductor. Since the power delivered by the current is related to the temperature distribution, temperature measurements for currents around $I_{0}=\bar{\tau }∆T/R$ can be used as the basis for a measurement technique of the Thomson coefficient. Equation (13) implies that the function P(I) represented in Figure 1 is the same as the function $\delta T(I)={T\left(L/2\right)-T}_{m}$ except for the factor 8K. Thus, experimental measurements of δT(I), around δT≈0, can be fitted to the second order polynomial $aI^{2}-bI$, where a and b are fitting parameters. The zero-power current, which corresponds to a sign reversal of $\delta T={T\left(L/2\right)-T}_{m}$, can be evaluated from the fitting parameters a and b as $I_{0}=b/a$. The Thomson coefficient can then be evaluated as $\tau =I_{0}R/\left(T_{h}-T_{c}\right)$.

**Figure 5:**
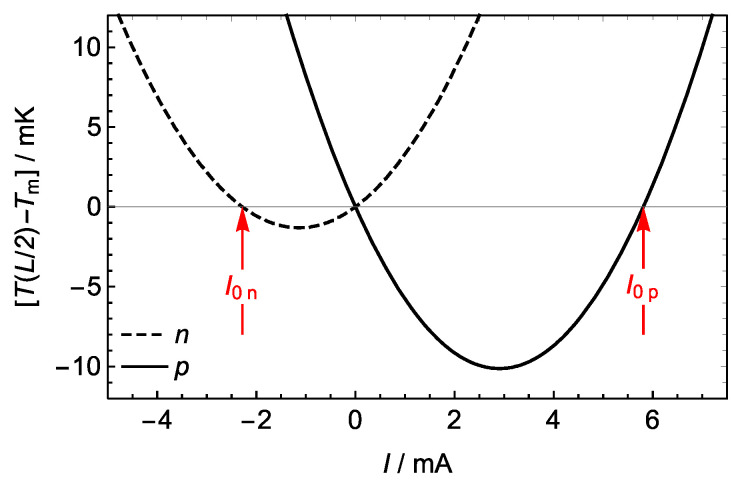
The difference $\delta T(I)=T\left(L/2\right)-T_{m}$ between the temperature at the center x=L/2 of a leg and the mean temperature $T_{m}=\left(T_{h}+T_{c}\right)/2$ has a parabolic dependence with the current I. The functions represented correspond to the *n*-type and *p*-type legs of the TEC described in Section 3.1. The zero-power currents $I_{0,n}$ and $I_{0,p}$ can be experimentally determined from the condition δT≈0, so that the average Thomson coefficient of each leg can thus be measured.

Alternatively, $I_{0}$ can be evaluated from the sign reversal of δT with varying I. In order to determine the accuracy needed in the measurement of this temperature difference, we can consider the TEs described in Section 3.1, with $\tau_{n}=-37.60\;\mu V\;K^{-1}$, $\tau_{p}=102.6\;\mu V\;K^{-1}$ and zero-power currents $I_{0,n}=-2.28\;mA$ and $I_{0,p}=5.81\;mA$. The functions $\delta T(I)={T\left(L/2\right)-T}_{m}$ for both legs have minima of −1.3 mK for the *n*-type and −10.1 mK for the *p*-type (Figure 5). Thus, we conclude that a null temperature detector with a sensitivity of 1 mK or lower allows for the accurate determination of $I_{0}$, and hence of the Thomson coefficient τ, from the sign reversal in $T\left(L/2\right)-T_{m}$ with varying I.

The zero-power current at which δT(I) reverses sign is not affected by the thermal conductivity of the material. However, for the same range of variation of the current, the values of δT(I) depend on the thermal conductivity. As described by Equation (13), the larger the thermal conductivity, the lower the values of δT(I) and the larger the uncertainty in the measured value of the Thomson coefficient. The 1 mK sensitivity of the temperature detector corresponds to ${Bi}_{2}{\left({Te}_{0.94}{Se}_{0.06}\right)}_{3\;}$ and ${{\left({Bi}_{0.25}{Sb}_{0.75}\right)}_{2}Te}_{3}$. Materials with lower conductivity are of interest to increase the figure of merit. The determination of $I_{0}$ and the Thomson coefficient would be more accurate in low thermal conductivity materials.

Note, finally, that the value of the Thomson coefficient that is determined with this method is that corresponding to the average of the hot and cold temperatures. The low currents required for the Thomson/Joule compensation ensure that the accuracy of the method is not sensitive to the temperature dependence of the material performance. Moreover, the use of a constant transport coefficients model with the values corresponding to this average temperature is further justified. 

## 4. Discussion and Conclusions

The Thomson effect in thermoelectric modules has been analyzed with special attention to its possible compensation with the Joule effect. The Thomson/Joule compensation ratio, the quotient between the negative Thomson power and the Joule power, has been studied both on global and local bases. The local Thomson/Joule compensation ratio is related to the curvature of the steady-state temperature distribution in the legs of a thermoelectric module.

The global Thomson/Joule compensation ratio is $I_{0}/I$. The compensation is exact when the electric current *I* equals the zero-power current $I_{0}$, i.e., due to the Thomson effect, the current absorbs the same power that it delivers due to the Joule effect. Since $I_{0}=\bar{\tau }∆T/R$ is proportional to the average Thomson coefficient, temperature measurements for currents around $I_{0}$ can be used to measure the Thomson coefficient. With varying current, the difference between the temperature at the center of the TE and the mean temperature between its extremes reverses its sign at $I=I_{0}$. Calculations using the constant transport coefficients model for ${Bi}_{2}{\left({Te}_{0.94}{Se}_{0.06}\right)}_{3\;}$ and ${{\left({Bi}_{0.25}{Sb}_{0.75}\right)}_{2}Te}_{3}$ led to the conclusion that a null temperature detector with a sensitivity of the order of 1 mK allows for the accurate determination of the Thomson coefficient. Reasonably, this result may stimulate new experimental studies.

## Data Availability

The original contributions presented in the study are included in the article, further inquiries can be directed to the corresponding author.

## Appendix A. On the Concept of “Heat Source”

The transport equations of thermoelectric phenomena relate the flux densities ${\{j}_{T},i\}$ and the generalized forces ${\{X}_{T},X_{e}\}$. The local rate of entropy production [37,47,48] is

(A1) $$\pi_{s}=\frac{1}{T}\left(j_{T}{\cdot X}_{T}+i{\cdot X}_{e}\right)=\frac{\kappa }{T^{2}}(\nabla T{)}^{2}+\frac{1}{T}\rho i^{2}.$$

The choice of $X_{T}=-(1/T)\nabla T$ as one of the forces is very convenient given the right-hand side of Equation (A1). Different choices of the “thermal” flux density $j_{T}$ and the “electrical” generalized force $X_{e}$ characterize the different possible formalisms used in thermoelectricity. Given their pivotal role, the observable formulation of thermoelectric phenomena chooses i and −∇T as the “independent” quantities that characterize the local non-equilibrium states.

The total energy flux density $j_{u}$ in Equation (2) is the sum of two terms: $j_{q}=-\kappa \nabla T$, due to thermal conduction, and another term that is the flux density of energy transported by the current, (Π−μ/e)i. The divergence of (Π−μ/e)i is the power delivered by the electric current (to the conductor). Similarly, the entropy flux density $j_{s}$ is the sum of two terms: one which is proportional to $X_{T}=-(1/T)\nabla T$, and another one which is the flux density of entropy transported by the current,

(A2) $$j_{s}=-\frac{\kappa }{T}\nabla T+Si.$$

Under steady-state conditions, the local balance equation for entropy is $\pi_{s}=\nabla \cdot j_{s}$. The right-hand side of Equation (A1) shows that the irreversible processes that produce entropy are thermal conduction due to a temperature gradient and conduction of electric current.

The concepts of “heat flux density” and “heat source” are not unique. Extensive state variables are those that can flow, such as electric charge, entropy, and energy. The expressions of their flux densities are undisputed. On the contrary, the heat flux density requires explaining the convention used, because the quantities describing thermodynamic processes, such as heat and work, do not flow. The convention used in Equation (3) is that we designate as heat flux density only the contribution to the energy flux density due to thermal conduction, i.e., $j_{q}=-\kappa \nabla T$. The electric current transports energy. One of the contributions to the energy transported by the current, Equation (4), is TSi=Πi. A usual convention in thermoelectricity is referring to *T*$j_{s}=-\kappa \nabla T+TSi$ as the “heat flux density”. In this convention, the divergence $\nabla \cdot (Tj_{s})$ is nonzero because of the local “heat production”. That is, the “heat sources” are considered to be the contributions to $\nabla \cdot (Tj_{s})$. From Equations (1) and (5), these “heat sources” are $\rho i^{2}$ and Si·∇T, because

(A3) $$\nabla \cdot (Tj_{s})=(1/e)i\cdot \nabla \mu =\rho i^{2}+Si\cdot \nabla T,$$

where we have used τ∇T=T∇S and ∇·i=0 [37]. Other authors consider as “heat sources” all the contributions to the negative divergence of the total energy flux density,

(A4) $$-\nabla \cdot j_{u}=\nabla \cdot \left(\kappa \nabla T\right)+\rho i^{2}-\tau i\cdot \nabla T,$$

that is, the Fourier, Joule, and Thomson terms, $\nabla \cdot \left(\kappa \nabla T\right)$, $\rho i^{2}$, and −τi·∇T [31]. Although these different conventions are all valid, the description of the Joule $\rho i^{2}$ and Thomson −τi·∇T contributions as *powers* delivered by the electric current seems to be sounder.

## Appendix B. Compensation in a Closed Circuit with No External Power Source

The concept of Thomson/Joule compensation can be applied to each one of the conductors composing a closed circuit, but not to the whole circuit because of the Peltier effect at the junctions. Consider a thermally insulated, closed circuit with no external power source that is only formed by two conductors, a and b, with Seebeck coefficients $S_{a}$ and $S_{b}(>S_{a})$, and with junction temperatures $T_{c}$ and $T_{h}(>T_{c})$. By closing the circuit, an electric current is established in the direction that tends to reduce the difference $T_{h}-T_{c}$ and drives the circuit towards thermal equilibrium. This direction corresponds to an increase in temperature in the conductor a, with a lower Seebeck coefficient. Since there is no external power source, there must be an internal source, the Seebeck power, which is negative in the closed circuit, $P_{S}=I\oint SdT<0$; note that $P_{S}>0$ when the current delivers power to the conductors, and $P_{S}<0$ when it absorbs power. The scalar product of Equation (1) with the current density i leads to ${(\rho /A)I}^{2}dx=(1/e)Id\mu -ISdT$, which can be integrated around the circuit to conclude that the Joule power $P_{J}=(I^{2}/A)\oint \rho dx>0$ is provided by the Seebeck power $P_{S}$,

(A5) $$P_{J}=-P_{S}\;(\mathrm{closed}\;\mathrm{circuit})$$

where we have used that the electrochemical potential μ of the electrons is a continuous function of position. That is, the Joule power $P_{J}$ delivered to the conductors by the current is the same as the power $-P_{S}$ absorbed from the conductors by the current due to the Seebeck effect. This Seebeck/Joule compensation is logical because the integration of Equation (1) clearly shows that the driving force for the electric current around the closed circuit is proportional to $\int_{T_{c}}^{T_{h}}\left(S_{b}-S_{a}\right)dT>0$. In the absence of energy exchange with the surroundings, the difference $T_{h}-T_{c}$ and the current I both tend to zero.

The energy flux density due to the Peltier effect is Πi. Thus, the power that enters a region V of a conductor due to this effect is $-{∯}_{\partial V}\Pi i\cdot dA=-{∭}_{V}i\cdot \nabla \Pi d^{3}r$ or, in one-dimensional form, $P_{\Pi }=-\int Id\Pi$. The scalar product of the equation −τ∇T=S∇T−∇Π with the current density i leads to

(A6) −IτdT=ISdT−IdΠ

Integration of Equation (A6) over a conductor

(A7) $$P_{T}=P_{S}+P_{\Pi }\;(\mathrm{conductor})$$

where $P_{T}=-I\int \tau dT$, $P_{S}=I\int SdT$ and $P_{\Pi }=-I\int d\Pi$ are the powers delivered by the current to the conductor due to the Thomson, Seebeck, and Peltier effects, respectively; negative values of any of these contributions represent absorbed power.

At the junctions between different conductors, the energy flux density $j_{u}$, Equation (2), is continuous but its Fourier and Peltier contributions, $j_{q}$ and Πi, show discontinuities (that cancel each other). Note that Equation (A6) is not valid at the junctions, where *T* is continuous but Π is not. For a closed circuit, −I∮dΠ=0. The power delivered by the current to the conductors a and b due to the Peltier effect is $P_{\Pi a}+P_{\Pi b}=-I\left(\Pi_{ah}-\Pi_{ac}\right)-I\left(\Pi_{bc}-\Pi_{bh}\right)$ and can equivalently be written as $P_{\Pi a}+P_{\Pi b}=-P_{\Pi h}{-P}_{\Pi c}=-I\left(\Pi_{ah}-\Pi_{bh}\right)-I\left(\Pi_{bc}-\Pi_{ac}\right)$. The quantity $P_{\Pi h}=I\left(\Pi_{ah}-\Pi_{bh}\right)<0$ can be considered as the power “delivered” to the hot junction due to the Peltier effect, and its logical negative sign means that the direction of the current is determined by the tendency to reach thermal equilibrium, reducing $T_{h}$; similarly, $P_{\Pi c}=I\left(\Pi_{bc}-\Pi_{ac}\right)>0$ to increase $T_{c}$. Note that $P_{\Pi h}$ and $P_{\Pi c}$ are the powers most often discussed in relation to the Peltier effect. Thus, adding the contributions of the two conductors, Equation (A7) can be transformed to

(A8) $$P_{S}=P_{T}+P_{\Pi h}{+P}_{\Pi c}\;(\mathrm{closed}\;\mathrm{circuit})$$

where $P_{S}=I\oint SdT=P_{Sa}+P_{Sb}$ and $P_{T}=-I\oint \tau dT=P_{Ta}+P_{Tb}$ are the Seebeck and Thomson powers delivered by the current to the conductors. Equation (A8) shows that, for the closed circuit, the Seebeck power includes the Thomson power and the Peltier powers “delivered” to the junctions [49]. Moreover, as shown in Equation (A5), the Seebeck power for the closed circuit is negative, $P_{J}=-P_{S}>0$. These results indicate that the concept of T/J compensation is useful for a conductor but not so much for a closed circuit.
